# A Visit to the Medical Association of the State of Alabama

**Published:** 1877-05

**Authors:** 


### A VISIT TO THE MEDICAL ASSOCIATION OF THE STATE OF ALABAMA.

Editor Chicago Medical Journal and Examiner:

Thinking the readers of the Journal and Examiner might chance to feel an interest in the condition of medical matters in the State of Alabama, as reflected through the constituted exponent of the profession—the Medical Association—I have taken a few pencil notes, which I offer for their acceptance.

This association was organized in the year 1840. With the exception of a few years when the chaos of social and business avocations incident to our late war, diverted the minds of men throughout the country from such pursuits, the annual meetings of the body have been held with regularity. As is usual with State societies, this of Alabama is migratory in its character, being carried in successive years from place to place amongst the larger towns of the State.

In its constituent membership it comprises a goodly number of names of men whose attainments and labors are matter of just pride, not only to Alabama, but to the South; names which do no discredit to the profession of the whole country. Were it not invidious to do so, I might mention a number of these names which would probably be familiar to the minds of your readers.

For several years past, the association has published an annual volume of transactions of creditable size, and of merit sufficient to draw forth the approval and warm commendation of leading medical journals all over the land. In this respect, at least, it may safely be said that no similar medical body in the South has surpassed it.

The association comprises in its membership all of the individual members of every county society in the State, who are privileged to attend the meetings and participate in its

*scientific* proceedings and discussions, but not in its ethical and business management. It is a representative body, and receives annually two delegates from each county society who compose the House of Delegates for the year, and who are entitled to vote upon matters of business which may be brought before the association. The House of Councilors consists of delegates usually, and sometimes other meritorious members, who may be elected by the house, to a number which shall not exceed one hundred in all; who are elected for life unless removed for cause, *e. g.*, non-payment of dues ($10 annually), absence from three consecutive sessions of the body, etc. The House of Councilors has especial charge of questions of ethics, and the general business policy of the association, and its members are alone eligible to hold office in the association. From the councilors is selected annually one member of the "Board of Censors," with a five year's tenure of office, and the five members constituting a medical court vested with absolute powers like those of the Judicial Council of the American Medical Association.

This association is constituted by law the State Board of Health, and a committee of the councilors, called the "Board of Health," is charged with this especial duty. The tenure of this office, like that of censor, is for five years, one new member being elected annually. At the present session the Board of Censors and the Board of Health have been consolidated into one body, charged in common with the duties of both, and bearing the name of censors. The machinery of the Health Board is not yet in full operation, but its foundations are being deeply and securely laid, and promise rich fruit in the near future.

At the last session of the State Legislature a law was enacted to regulate the practice of medicine and surgery in the State, and the State Medical Association was charged with its execution. Under this law, those who offer for practice of regular medicine in the future, are to be examined upon all of the branches, whilst irregulars are to be subjected to examination only in anatomy, physiology, chemistry, surgery, and obstetrics. These examinations are to be made of all applicants, whether graduates or not. Those candidates who have diplomas are examined by the Boards of their several counties, whilst all who are without diplomas must appear before the Board of Censors of the State Association, which is constituted the State Board of Medical Examiners. It is required that all who are now practicing in the State shall appear before their county Boards and take out certificates of registration without examination. These certifieates are to be recorded in the office of the Probate Court, and the record renewed when the residence is changed from one county to another.

At the present session of the association the attendance is smaller than usual, on account of a recent flood in the water courses obstructing travel. A number of valuable papers were read, but the scientific features of the proceedings were hardly so sumptuous as last year. A marked feature of this body is the regular attendance of members from different parts of the State, year after year, evidencing the interest in its welfare, which is felt, and a readiness to make some sacrifices in order to be present at its meetings. The average intellectual capacity of the members, and their professional attainments, strike a visitor as being highly creditable; this is probably due to the fact that the meetings are filled up by earnest men from all quarters of the State, rather than by local practitioners, who attend because of the close proximity of the meeting to their places of residence.

ROBERT BATTY, M. D.

ROME, GA., April, 1877.

---

### THE LATE DR. FREER.

TO THE EDITOR OF THE JOURNAL AND EXAMINER:

*Dear Sir:*—Among all the high eulogies so deservedly bestowed upon the late Dr. Freer, the writer finds no mention of a certain phase of his character which is deserving the highest praise, viz., the honorable position he took in regard to medical women. He was not only gentlemanly toward them but just, seeming to regard them in every respect as his peers.

It is hoped you will pardon the writer as she gives a personal experience in regard to this point, for outside of the faculty of the Woman's College she has found no such friend in the profession as Dr. Freer. The first time she ever saw him he extended to her the unprecedented courtesy of inviting her to a place beside him at the operating table during his surgical clinic. Just then, entering upon a new and untried field, she expressed to him the hesitancy and dread with which she undertook the work—the teaching of physiology, his own branch, and asked him for advice. He replied: "You do not need advice, you will be sure to succeed, and your first course of lectures will be your best. I think mine was, because I worked the hardest over it." He then offered her the use of any material at his disposal, and said he would be glad to give any assistance in his power. A year since, he transferred to her his appointment as a delegate from the State Medical Society to the American Medical Association—the result of which you well know—and the kind letter he wrote on that ocasion is preserved as a precious memorial. The last time the writer ever saw him was at the opening (or dedication) of Rush College. While conversing with him at the close of the exercises, she spoke of the future of the Woman's College, and remarked that the great disparagement between his college with its magnificent new building, and ours with no building at all, was enough to discourage one. He replied: "Not at all. You must never abandon that college; sooner or later it is bound to be a success. If you give it up, it is only to give it into the hands of others; a woman's college in Chicago is a fixed fact, and I am determined that Rush College shall befriend you in every way possible." Such a sentiment from such a man is most worthy of record. May the successor of this great man be as noble and as just.

SARAH HACKETT STEVENSON, M. D.

*Prof. of Physiology Woman's Hosp. Med. College.*

ST. CAROLINE'S COURT, April 18, 1877.

